# Sudden restoration of the band ordering associated with the ferromagnetic phase transition in a semiconductor

**DOI:** 10.1038/ncomms12013

**Published:** 2016-06-28

**Authors:** Iriya Muneta, Shinobu Ohya, Hiroshi Terada, Masaaki Tanaka

**Affiliations:** 1 Department of Electrical Engineering and Information Systems, The University of Tokyo, 7-3-1 Hongo, Bunkyo-ku, Tokyo 113-8656, Japan; 2 Center for Spintronics Research Network, Graduate School of Engineering, The University of Tokyo, 7-3-1 Hongo, Bunkyo-ku, Tokyo 113-8656, Japan

## Abstract

The band ordering of semiconductors is an important factor in determining the mobility and coherence of the wave function of carriers, and is thus a key factor in device performance. However, in heavily doped semiconductors, the impurities substantially disturb the band ordering, leading to significant degradation in performance. Here, we present the unexpected finding that the band ordering is suddenly restored in Mn-doped GaAs ((Ga,Mn)As) when the Mn concentration slightly exceeds ∼0.7% despite the extremely high doping concentration; this phenomenon is very difficult to predict from the general behaviour of doped semiconductors. This phenomenon occurs with a ferromagnetic phase transition, which is considered to have a crucial role in generating a well-ordered band structure. Our findings offer possibilities for ultra-high-speed quantum-effect spin devices based on semiconductors.

## Abstract (web summary)

As semiconductors are doped with impurities, their useful electrical transport properties are degraded as their band structures are increasingly modified. Here, the authors demonstrate that the band ordering is restored in Mn-doped GaAs above a ferromagnetic transition at a critical concentration.

## Graphical abstract

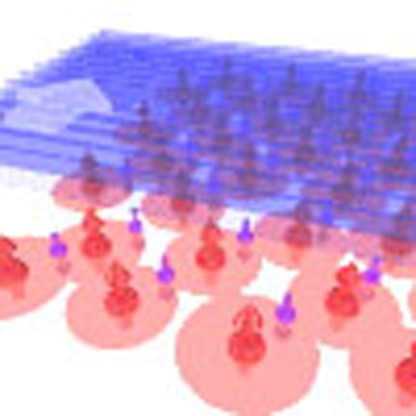

In semiconductor devices, the mobility or the coherence of the wave function is a key factor in determining the device performance, and obtaining a well-ordered band structure is quite important for developing these characteristics. Much effort has been made for this purpose in the long history of semiconductor research. Especially, the modulation doping technique^1^, where dopants are placed away from the carrier transport channel to reduce impurity scattering, has successfully led to dramatic enhancement of the carrier mobility, and it is indispensable for the present high-speed electronic devices^2^. To obtain even higher carrier mobility and better device performance, many attempts have been made, such as strain control^3^, improvement of the interface flatness and quality^4^, and further purification of the carrier transport channel^5^. However, almost all these studies have been carried out based on the common understanding that an increase in the impurity concentration just disturbs the band ordering and thus reduces the mobility or the coherence of the wave function of carriers. This is one of the biggest well-known dilemmas that have been hardly doubted in the research of semiconductors.

In this article, we find unexpected and opposite behaviour that the band ordering is restored by increasing the doping concentration of magnetic impurities in a semiconductor when it experiences a ferromagnetic phase transition that is induced by the doping. Our result may open up a way to control the band ordering by using doping and a phase transition.

## Results

### Samples

**Figure 1:**
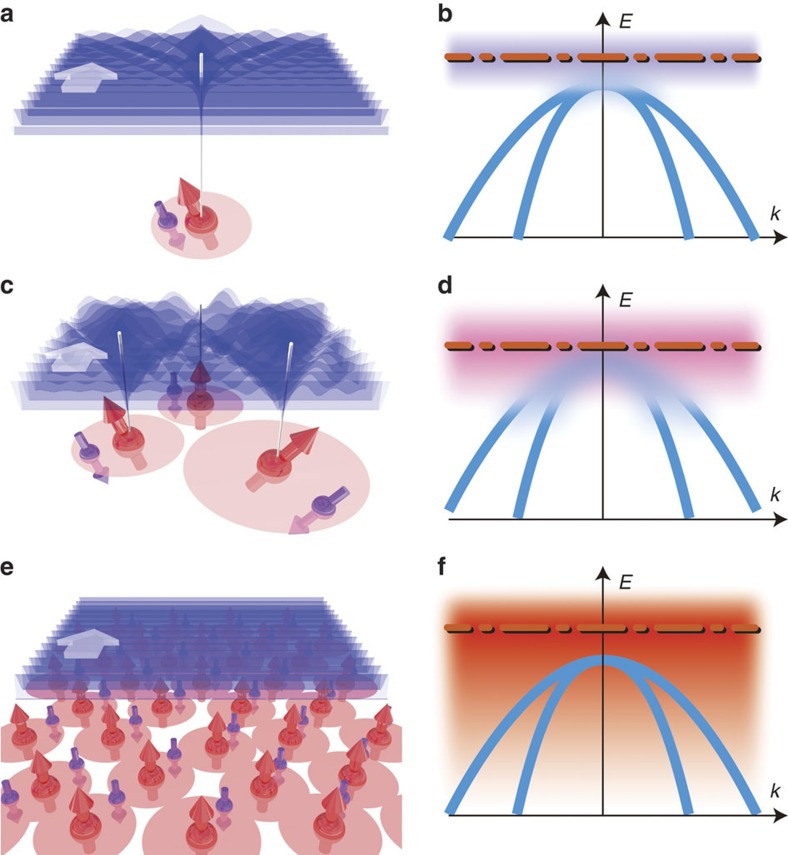
Schematic illustration of our finding showing that the VB ordering is restored with the ferromagnetic transition in Mn-doped GaAs. (**a**,**c**,**e**) Schematic views of the plane-wave envelope function of the Bloch function of the VB holes (blue waves), the localized Mn *d* spins (large red spins) and the localized *p*-holes (small spins), which are antiferromagnetically coupled with the Mn *d* spins, when GaMnAs is in the paramagnetic state (**a**), in the paramagnetic state just before the ferromagnetic transition (**c**) and in the ferromagnetic state (**e**). (**b**,**d**,**f**) VB dispersion curves (blue curves) and the IB (dispersionless horizontal bands) when GaMnAs is in the paramagnetic state (**b**), in the paramagnetic state just before the ferromagnetic transition (**d**) and in the ferromagnetic state (**f**). Here, the red dash-dotted lines represent the Fermi level. In the paramagnetic state, the wave function of the VB holes is disturbed by the Mn impurity atoms (**a**,**c**), and thus, the VB ordering is disturbed (**b**,**d**). In the ferromagnetic state, the wave function of the VB holes becomes insensitive to the fluctuations in the IB (or to individual Mn atom) (**e**) and can coherently move in the crystal, thus establishing a well-ordered VB (**f**).

**Figure 2:**
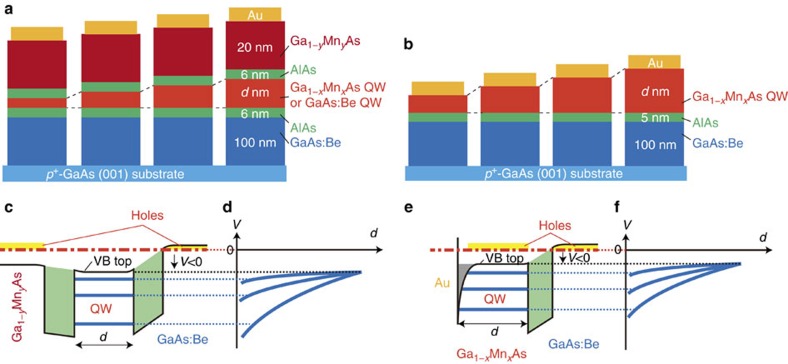
Schematic device structure and the principle of resonant tunnelling spectroscopy. (**a**,**b**) The device structures investigated in this study are composed of Ga_1−*y*_Mn_*y*_As (20 or 50 nm)/AlAs (6 nm)/Ga_1−*x*_Mn_*x*_As QW or GaAs:Be QW (*d* nm)/AlAs (6 nm)/GaAs:Be (100 nm) on a *p*^+^GaAs (001) substrate for samples A–M and P–Y (**a**) and GaMnAs QW (*d* nm)/AlAs (5 nm)/GaAs:Be (100 nm) on a *p*^+^GaAs (001) substrate for samples O1–O3 (**b**). After the growth, we fabricated circular mesa diodes of 200 μm in diameter. In samples A–L, O1–O3, P and S–Y, the QW thickness *d* varies in the ranges shown in Table 1 on the same wafer, whereas *d* is fixed for samples M, Q and R. The hole concentrations in the GaAs:Be electrode and the GaAs:Be QW are ∼1 × 10^18^ cm^−3^ and ∼1 × 10^19^ cm^−3^, respectively. (**c**,**e**) Schematic VB diagrams of the DB-QW heterostructure with a GaMnAs QW (**c**) and the single-barrier heterostructure with a GaMnAs electrode (**e**). The black solid curves (or lines), the thick blue lines and the red dash-dotted line represent the VB top energy, the quantum levels and the Fermi level, respectively. (**d**,**f**) Idealized plot of the bias voltages *V* that correspond to the resonant levels as a function of *d*, which converge to the bias voltage that corresponds to the VB top energy of the QW with increasing *d*. Zero bias corresponds to the Fermi level. If the converged voltage is in the negative-bias region, as is the case for GaMnAs QWs, the Fermi level exists in the band gap.

To demonstrate the doping concentration dependence of the band ordering as schematically shown in Fig. 1, we used the model-system ferromagnetic-semiconductor Mn-doped GaAs (Ga_1−*x*_Mn_*x*_As)^6^,^7^,^8^ in which the Mn atoms partially replace the Ga sites and act as both localized spins and acceptors. Ferromagnetism appears at low temperature when the Mn concentration *x* is > ∼0.9%. We have performed resonant tunnelling spectroscopy^9^ on a series of double-barrier (DB) heterostructures with a Ga_1−*x*_Mn_*x*_As quantum well (QW) with *x* ranging from 0.03% to 2.3% (Table 1). The Ga_1−*x*_Mn_*x*_As QW layer is paramagnetic when *x*<∼0.9% and ferromagnetic when *x*≥∼0.9%. The examined DB-QW heterostructures, denoted as samples A–M and S–Y, are composed of Ga_1−*y*_Mn_*y*_As (20 or 50 nm)/AlAs (6 nm)/Ga_1−*x*_Mn_*x*_As QW (*d* nm)/AlAs (6 nm)/GaAs:Be (100 nm) grown on a *p*^+^GaAs (001) substrate (Fig. 2a). As references, we prepared samples P–R, which have the same DB-QW structure but a degenerate Be-doped *p*^+^-GaAs QW, where the Be concentration is ∼1 × 10^19 ^cm^−3^, instead of the GaMnAs QW. During the growth of the QW layers in samples A–L, S–Y and P, we moved the main shutter in front of the wafer such that we obtained many mesa diodes with various QW thicknesses *d* on the same substrate (see Methods section). The Mn content *y* of the top Ga_1−*y*_Mn_*y*_As electrode was fixed at 4–6%. In addition, we have performed resonant tunnelling spectroscopy on a single-barrier heterostructures with a GaMnAs electrode (samples O1–O3), which are composed of Ga_1−*x*_Mn_*x*_As (*d* nm)/AlAs (5 nm)/GaAs:Be (100 nm) grown on a *p*^+^GaAs (001) substrate (Fig. 2b). In the single-barrier heterostructures, the valence-band (VB) holes are confined between the AlAs barrier and the Schottky barrier formed at the surface of the GaMnAs layer, and thus the quantum levels are formed in the GaMnAs layer^10^ (Fig. 2e). We measured the tunnel current *I* at 3.5 K by applying a bias voltage *V* to the top of the mesa diodes and grounding the bottom of the wafer. As illustrated in Fig. 2c,e, when a negative-bias voltage is applied, holes are injected from the GaAs:Be electrode, which has a small Fermi surface, into the small in-plane wave-vector region in the QW, and thus, each resonant tunnelling level can be detected separately^9^,^10^,^11^,^12^ (See Supplementary Note 1). With increasing *d*, these resonant levels converge to a bias voltage that corresponds to the VB top energy of the QW material, where zero bias corresponds to the Fermi level. For example, when the Fermi level exists in the band gap, as is the case for GaMnAs, the converged bias voltage is negative (Fig. 2d,f). We note that nearly the highest limit of the growth temperature *T*_S_ was used for the growth of the GaMnAs QW to obtain a high-quality QW in all samples used in this study (Supplementary Fig. 1; Supplementary Note 2) except for sample U (Supplementary Note 3; Supplementary Fig. 2). In fact, the Curie temperatures (*T*_C_) of the GaMnAs QW in these samples follow the *T*_C_–*x* curve obtained for high-quality GaMnAs samples reported in ref. 13 (Supplementary Fig. 3).

### Resonant tunnelling experiments

By carefully comparing the resonant tunnelling spectroscopy results of the devices with the non-magnetic GaAs:Be QW (samples P, Q and R) as references, we have confirmed that the d^*2*^*I*/d*V*^*2*^ oscillations observed in the GaMnAs-QW heterostructures discussed later are not induced by the resonant levels formed in the triangular potential region at the AlAs/GaAs:Be interface^14^ or by the diffused Mn or Be atoms in the AlAs barriers, but are definitely induced by the resonant levels formed in the GaMnAs QW (Supplementary Note 4; Supplementary Fig. 4).

**Figure 3:**
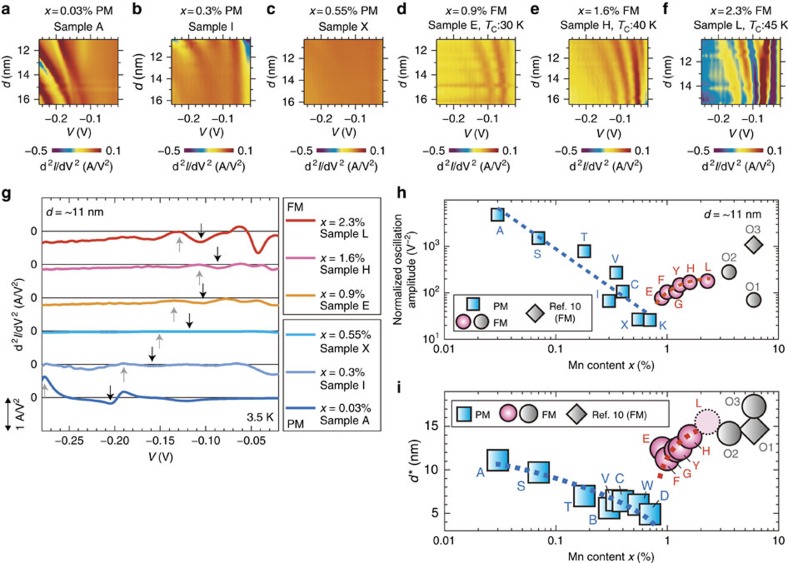
Results of the estimation of the *x* dependence of the band ordering. (**a**–**f**) Colour-coded maps representing d^*2*^*I*/d*V*^*2*^ as a function of the QW thickness *d* and the applied bias voltage *V* for the Mn concentration *x* values of 0.03, 0.3, 0.55, 0.9, 1.6 and 2.3%. The oscillations become weaker as *x* increases from 0.03% to 0.55%, but are suddenly restored after the ferromagnetic transition (*x*≥∼0.9%). (**g**) d^*2*^*I*/d*V*^*2*^-*V* characteristics of samples L, H, E, X, I and A at 3.5 K when *d* is fixed at ∼11 nm. The black and grey arrows indicate the third HH dip and peak, respectively. (**h**) Normalized oscillation amplitude defined as eq. (1) as a function of *x*. (**i**) d* as a function of *x*, where d* is defined as the *d* value at which the peak between HH1 and the first light hole dips disappears with increasing *d*. The rectangular and circular points represent the data values of the paramagnetic (PM) and ferromagnetic (FM) samples, respectively. As a reference, we show the d* value obtained for the single-barrier heterostructures (samples O1–O3, *x*=3.6–6%) with a GaMnAs surface QW (grey circles) and in ref. 10 (a grey diamond); these reference values indicate that the VB ordering remains high even for such a high Mn content of 6%. For sample L (*x*=2.3%), we plot the lower limit of d* (light pink dot with a broken circle), because d* is estimated to be over the *d* range measured on sample L. (The data of samples H, I and L are the same as those in ref. 15).

In all the samples with the Ga_1−*x*_Mn_*x*_As QW, we have observed clear d^*2*^*I*/d*V*^2^ oscillations induced by resonant tunnelling in the QW, regardless of the value of *x* (0.03–2.3%) (Fig. 3a–f, and also see Supplementary Figs 5–7). The observed resonant levels in the d^2^*I*/d*V*^2^ oscillations correspond to the quantum levels in the GaMnAs QW. In the negative bias, the dips in the d^2^*I*/d*V*^2^ oscillations represent the resonant levels. As *d* increases, the resonant levels are converging to the certain voltage, whose behaviour is ascribed to the *d* dependence of the quantum levels as illustrated in Fig. 2d,f (see Supplementary Note 5 and Supplementary Fig. 7 for more detail about the comparison of the measured and calculated resonant levels). The key observation in Fig. 3a–f is that the intensity of the oscillations changes depending on *x*. The oscillation intensity becomes weaker when *x* is increased from 0.03% to 0.55% in the paramagnetic region, which indicates that the band structure becomes disordered, as in other semiconductors that are heavily doped with non-magnetic impurities. However, in the ferromagnetic region for *x*≥0.9%, the oscillations are restored at *x*=0.9%, and their intensity gradually increases with increasing *x*, which indicates that the band order is first restored and then improved with increasing *x*. The sudden restoration of the band order is atypical of the general behaviour of doped semiconductors (Supplementary Note 6; Supplementary Table 1; Supplementary Figs 8 and 9) and can be considered to be related to the ferromagnetic transition that occurs at precisely the same time (*x*=0.9%).

For easier understanding of the *x* dependence of resonant tunnelling, we quantify the oscillation amplitude of the d^2^*I*/d*V*^2^−*V* curves of the samples with various *x* ranging from 0.03% to 2.3%, when *d* is fixed at 11 nm (Fig. 3g) by defining the normalized oscillation amplitude of these d^2^*I*/d*V*^2^−*V* characteristics as


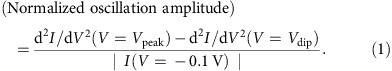


Here, *V*_dip_ and *V*_peak_ are the voltages of the HH3 dip and peak of the d^2^*I*/d*V*^2^−*V* curves, which are shown as the black and grey arrows in Fig. 3g, respectively. (Here, HH3 means the heavy hole (HH) third level in the GaMnAs QW. See Supplementary Fig. 7 for the assignment of the resonant levels.) The normalization (denominator) is necessary to eliminate the influence of the difference in the tunnelling resistance depending on the samples. As shown in Fig. 3h, the normalized oscillation amplitude decreases as *x* increases in the paramagnetic region of *x*<0.9%, but it suddenly increases in the ferromagnetic region of *x*≥0.9%.

Moreover, to quantitatively evaluate the VB ordering, we define d* as the *d* value at which the peak between the dips of the HH first level and the light hole first level disappears (see Supplementary Note 7 and Supplementary Figs 10–12 for more detailed description of the determination of d*). The band ordering is defined as the spatial ordering of the wave functions of holes. A highly ordered band is necessary for the formation of quantum levels in a QW and thus for the resonant tunnelling effect. If the resonant tunnelling is weak (strong), the dips of the resonant oscillation are broad (sharp) and they merge at small (large) *d*. Thus, d* is a good indicator for the VB ordering. Because the VB holes must make a round trip in the QW for the formation of the quantum levels, 2d* corresponds to the coherence length of the VB holes. The mobility *μ* is expressed by *qτ*/m*, where *q* is the elementary charge, τ is the relaxation time of holes and m* is the effective mass of the VB holes in GaMnAs, which is thought to be almost the same as that of GaAs. Because *τ* is proportional to the coherence length, *μ* is proportional to the coherence length. Therefore, the experimentally estimated d* values represent the VB ordering, coherence length and mobility. As shown in Fig. 3i, d* decreases as *x* increases in the paramagnetic region, but there is a sharp jump in d* at the onset of ferromagnetism (*x*=0.7–0.9%). Both the normalized oscillation amplitude and d* (Fig. 3h,i) show the same behaviour. This clearly indicates that the VB becomes disordered as *x* increases in the paramagnetic region; however, the VB ordering is restored at the onset of the ferromagnetic transition.

One may think that the resistance area difference between the devices may influence the intensity of these oscillations, but there is no clear correlation between the resistance area value and d* (Supplementary Note 8; Supplementary Fig. 13). Also, we do not observe any sudden change in the crystallinity at *x*=0.7–0.9%, either in the data of the hole concentration versus *x* or in the data of the film conductivity versus *x* (Supplementary Note 9; Supplementary Fig. 14). Therefore, the sudden restoration of the VB ordering is intrinsic to GaMnAs. (In addition, we note that the Fermi level position of the samples containing the GaMnAs QW, which was estimated in our previous studies, moves continuously by increasing *x* (ref. 15), but this *x* dependence of the Fermi level position is not directly related to the sudden restoration of the VB ordering observed in the present study (Supplementary Note 9)).

### Temperature dependence

**Figure 4:**
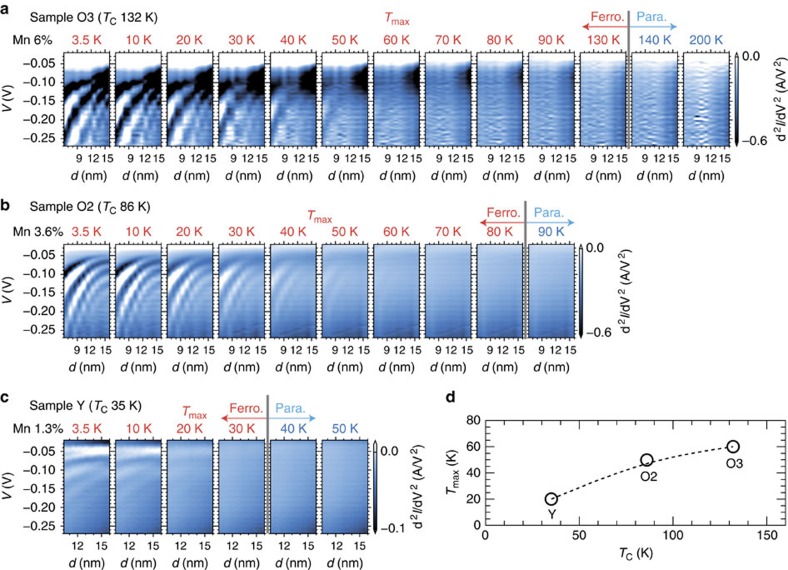
Comparison of the temperature dependence of the resonant tunneling characteristics among the samples with different *x* and *T*_C_. (**a**–**c**) Colour-coded maps representing d^2^*I*/d*V*^2^ as a function of the QW thickness *d* and the applied bias voltage *V* in sample O3 (the Mn concentration *x*: 6%, the Curie temperature *T*_C_: 132 K, *T*_max_: 60 K) (**a**), sample O2 (*x*: 3.6%, *T*_C_: 86 K, *T*_max_: 50 K) (**b**) and sample Y (*x*: 1.3%, *T*_C_: 35 K, *T*_max_: 20 K) (**c**). (**d**) Relation between *T*_C_ and *T*_max_. The characters in the graph represent the names of our samples.

To confirm the strong relation between the resonant tunnelling and the ferromagnetic ordering in GaMnAs, we measured the temperature (*T*) dependence of resonant tunnelling for three samples with different Mn content *x* and different *T*_C_; samples Y (*x*=1.3%, *T*_C_=35 K), O2 (*x*=3.6%, *T*_C_=86 K) and O3 (*x*=6%, *T*_C_=132 K), as shown in Fig. 4a–c. In Fig. 4, the oscillation is not observed in the d^2^*I*/d*V*^2^−*V* characteristics in the paramagnetic region (*T*>*T*_C_) for all the samples. In the ferromagnetic region (*T*<*T*_C_), the oscillation induced by resonant tunnelling appears in the d^2^*I*/d*V*^2^−*V* characteristics as *T* decreases. Moreover, the oscillation amplitude is enhanced as *T* decreases. We define *T*_max_ as the maximum temperature at which we can observe resonant tunnelling with multiple dips in the d^2^*I*/d*V*^2^−*V* curves (see Supplementary Note 10 and Supplementary Figs 15–17 for the estimation of *T*_max_). As seen in Fig. 4d, *T*_max_ increases as *T*_C_ increases. Thus, these experiments also provide important evidence that the resonant tunnelling (thus the VB ordering) is correlated to the ferromagnetic ordering.

## Discussion

We discuss the mechanism underlying the change of the band ordering in GaMnAs when the film is changed from paramagnetic to ferromagnetic. In the paramagnetic region (*x*<∼0.9%), the Mn impurities induce a Coulomb potential, which binds the holes and produces the paramagnetic impurity band (IB) (Fig. 1a). As *x* increases, this Coulomb potential is screened, and thus, the impurity states become shallow and extended (Fig. 1c). This extended potential causes a fluctuation in the VB near the **Γ** point because of the extended nature of the states. Thus, the VB merges with the paramagnetic IB and becomes disordered (Fig. 1b,d), quite similar to the situation found in general semiconductors doped with non-magnetic impurities.

In the ferromagnetic region (*x*≥∼0.9%), however, the VB ordering is restored, which means that VB escapes the influence of the fluctuation of IB. This phenomenon cannot be explained by the conventional VB conduction picture of the band structure in GaMnAs, although it has been widely believed that VB and IB are merged, which yields a weakly disordered spin-split VB^16^. The sharp restoration of the VB ordering observed here is considered to be related to the complete screening of the Coulomb potential^15^ and the consequent emergence of a strong *p–d* exchange interaction involved with the *p*–*d* hybridization, which generates a disordered IB around the Fermi level^17^,^18^,^19^,^20^,^21^,^22^,^23^ (Fig. 1e,f). Indeed, recent photoemission experiments have revealed a large *p*–*d* hybridization energy as high as 1–2.5 eV in GaMnAs^24^,^25^,^26^. The Anderson impurity model^27^, which is a basic theory used for describing the hybridized bands in heavy fermion systems^28^, is appropriate for discussing the band structures strongly influenced by the *p*–*d* hybridization in GaMnAs. The Anderson impurity model in transition-metal-doped semiconductors has been theoretically developed^29^,^30^, and in the GaMnAs case, the strong exchange interaction induces localized impurity states^17^,^23^, which do not merge with the VB because of their localized nature. Thus, the disordered IB and the highly ordered VB suddenly manifest themselves with the ferromagnetic transition, as shown in Fig. 1f.

It will be worthwhile to note the similarity of band structures between heavy fermion materials (HFMs) and GaMnAs, even though it is difficult to apply the periodic Anderson model to GaMnAs just as in HFMs such as rare-earth compounds (for example, CeAl_3_ and UPt_3_), because Mn atoms in GaMnAs are dilute and distributed randomly. In HFMs, the large hybridization between orbitals yields a heavy band strongly influenced by the hybridization and a light band that is the same as the host band structure. Similarly, the large hybridization between As 4*p* and Mn 3*d* orbitals in GaMnAs is expected to yield two different bands; the localized disordered IB strongly influenced by the hybridization and the disorder induced by the random distribution of the Mn atoms, and the host-like highly ordered VB that is reflecting the perfect periodicity of the semiconductor lattice. The As 4*p* and Mn 3*d* orbitals are strongly hybridized, but the yielded band structures are separated to a localized band and an extended band.

We note that recent angle-resolved photoemission spectroscopy measurements of GaMnAs^31^,^32^ have given evidence of a well-ordered VB and the undisturbed *k*-dispersion of the VB, which are consistent with our results. Our finding will be useful for establishing ferromagnetism in semiconductors without disturbing the band ordering of the host semiconductor, which is especially promising for quantum-effect spin devices, where high coherence of the carrier wave function and ferromagnetism are simultaneously desired.

## Methods

### Heterostructure growth

For samples A–M and P–Y, we grew the DB-QW heterostructures (Table 1; Fig. 2a) using molecular-beam epitaxy (MBE), in which the GaAs:Be electrode, bottom AlAs and top Ga_1−*y*_Mn_*y*_As layers were grown at 600 °C, 560 °C and 210–220 °C, respectively. In each device structure, we grew the top AlAs layer at the same *T*_S_ used for the Ga_1−*x*_Mn_*x*_As QW or for the GaAs:Be QW (Table 1). In samples A–L, S–Y and P, we moved the main shutter in front of the substrate during the growth of the QW layer to produce a range of *d* values on the same wafer. We estimated the *d* values based on the speed of the main shutter. For samples O1–O3, we grew single-barrier heterostructures with a GaMnAs electrode (Fig. 2b) using MBE in which the GaAs:Be and AlAs layers were grown at 600 and 560 °C, respectively (see Table 1 for the *T*_S_ values of the surface Ga_1−*x*_Mn_*x*_As layer). Samples O1–O3 were annealed after the growth to improve the *T*_C_ value of the GaMnAs layer.

### Lithographic process

After the MBE growth, we fabricated circular mesa diodes of 200 μm in diameter using photolithography and chemical wet etching with phosphoric acid and hydrogen peroxide. In addition, we carefully etched the surface of each mesa diode by 1–3 nm to remove the surface oxide for samples A–M and P–Y. For samples O1–O3, to prepare tunnel junction devices with various *d* values on the same wafer, we vertically and carefully sink the wafer into the etchant^10^,^12^. We coated the sample with an insulating resist layer, made a circular contact hole with a diameter of 180 μm on top of each mesa, and fabricated a gold electrode for each mesa diode.

### Measurements

We applied a bias voltage *V* between the top and bottom electrodes and measured the tunnel current *I* while grounding the bottom electrode. We numerically obtained the d^*2*^*I/*d*V*^*2*^−*V* characteristics from the measured *I*–*V* characteristics; the Savitzky–Golay method was used for differentiation.

### Data availability

The data that support the findings of this study are included in Supplementary Information, and other data are available from the corresponding authors upon request.

## Additional information

**How to cite this article:** Muneta, I. *et al.* Sudden restoration of the band ordering associated with the ferromagnetic phase transition in a semiconductor. *Nat. Commun.* 7:12013 doi: 10.1038/ncomms12013 (2016).

## Supplementary Material

Supplementary Information Supplementary Figures 1-17, Supplementary Table 1, Supplementary Notes 1-10 and Supplementary References

## Figures and Tables

**Table 1:** Details of the samples investigated in this study.

**Sample**	**Structure**	***x*** **(%)**	***y*** **(%)**	***d*** **(nm)**	***T***_**S**_ **(°C)**	***T***_**C**_ **(K)**
A	Double barrier	0.03	6	11–16	400	Paramagnetic
B*	Double barrier	0.3	6	4–10	330	Paramagnetic
C	Double barrier	0.4	6	5–11	320	Paramagnetic
D	Double barrier	0.7	6	1–6	300	Paramagnetic
E	Double barrier	0.9	6	11–16	270	30
F*	Double barrier	1.0	6	10–16	265	25
G*	Double barrier	1.2	6	10–16	260	30
H*	Double barrier	1.6	6	10–16	250	40
I*	Double barrier	0.3	6	10–16	330	Paramagnetic
K	Double barrier	0.7	6	11–13	280	Paramagnetic
L*	Double barrier	2.3	6	10–16	240	45
M	Double barrier	1.0	6	100	270	Paramagnetic
O1^†^	Single barrier	6	—	1–19	210	111
O2	Single barrier	3.6	—	6–16	235	86
O3	Single barrier	6	—	6–16	220	132
P	Double barrier	Be-doped	5	5–18	500 & 400	Non-magnetic
Q	Double barrier	Be-doped	4	12	350	Non-magnetic
R	Double barrier	Be-doped	4	12	300	Non-magnetic
S	Double barrier	0.07	6	6–12	340	Paramagnetic
T	Double barrier	0.18	6	4–10	320	Paramagnetic
U	Double barrier	0.3	6	4–10	265	Paramagnetic
V	Double barrier	0.35	6	4–11	300	Paramagnetic
W	Double barrier	0.55	6	3–8	290	Paramagnetic
X	Double barrier	0.55	6	10–16	290	Paramagnetic
Y	Double barrier	1.3	6	10–16	265	35

The structures of our samples, Mn contents *x* of the Ga_1−*x*_Mn_*x*_As QW and *y* of the top Ga_1−*y*_Mn_*y*_As electrode, the QW thickness *d*, the growth temperature *T*_S_ of the QW layer and the Curie temperature *T*_C_ of the Ga_1−*x*_Mn_*x*_As QW. The hole concentration in the GaAs:Be electrode and in the GaAs:Be QW are ∼1 × 10^18^ cm^−3^ and ∼1 × 10^19^ cm^−3^, respectively. The sample names indicated with marks (* and †) are the same samples used in our previous paper, refs 15, 10, respectively. During the growth of the GaAs:Be QW in sample P, the first 5-nm-thick flat QW layer was grown at 500 °C. Then, the wedge-shaped QW layer was grown at the relatively low temperature of 400 °C to prevent the re-evaporation of the As atoms from the surface because no As flux was supplied to the surface area covered by the main shutter, which was moved during the growth of the QW layer.
