# Supplementary material for: Sudden restoration of the band ordering associated with the ferromagnetic phase transition in a semiconductor
Source: Nat Commun. 2016 Jun 28;7:12013. doi: 10.1038/ncomms12013 (PMC4931231; doi:10.1038/ncomms12013)
Supplement: Supplementary Information — Supplementary Figures 1-17, Supplementary Table 1, Supplementary Notes 1-10 and Supplementary References [file ncomms12013-s1.pdf]

Supplementary Figures

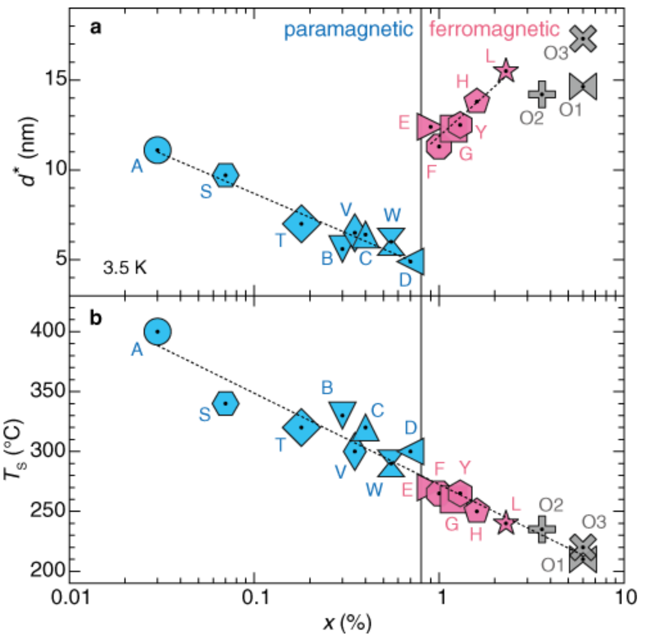

Supplementary Figure 1 Plots of d^* and the growth temperature as a function of x. (**a**) d^* as a function of the Mn concentration x. We define d^* as the quantum well thickness d value at which the peak between the dips of the first heavy and light hole levels in the d^2I/dV^2-V characteristics disappears as d increases. (**b**) Growth temperature T_S during the molecular-beam epitaxy growth of our $Ga_{1-x}Mn_xAs$ layers plotted as a function of x. The blue and pink symbols represent the paramagnetic and ferromagnetic samples, respectively. The gray symbols represent the single barrier samples. The characters in the figures are the sample names.

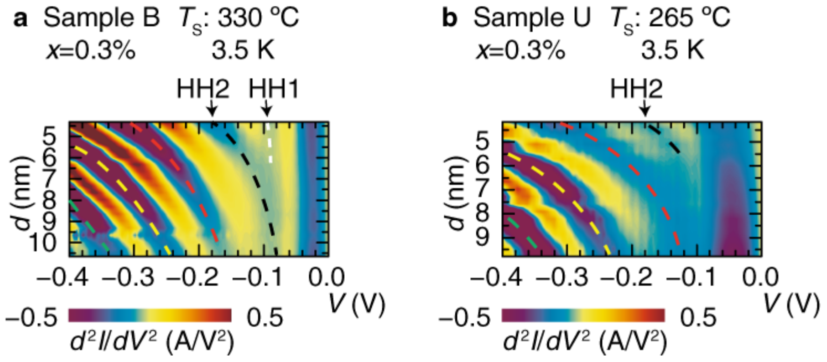

Supplementary Figure 2 Comparison of the d^2I/dV^2-V characteristics between the samples grown at different growth temperatures. (**a**,**b**) Colour-coded map representing d^2I/dV^2 as a function of the quantum well thickness d and the applied bias voltage V in Sample B (the Mn concentration x: 0.3%, the growth temperature T_S: 330 °C) (**a**) and Sample U (x: 0.3%, T_S: 265 °C) (**b**) at 3.5 K. The white, black, red, yellow and green dashed lines represent the first heavy hole (HH), HH2 [and first light hole (LH)], HH3, HH4 (and LH2) and HH5 resonant dips, respectively.

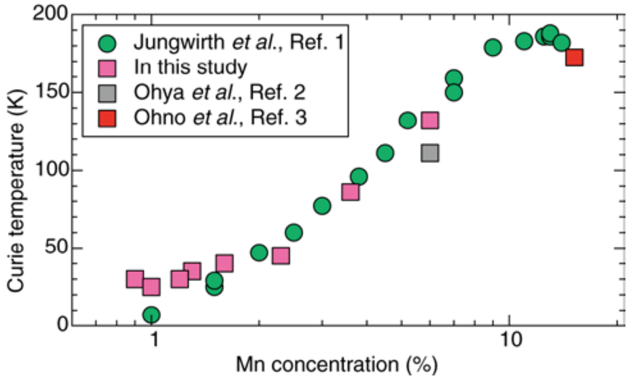

Supplementary Figure 3 Curie temperature as a function of the Mn concentration in GaMnAs. The circles and squares represent the GaMnAs samples grown by Jungwirth *et al.*[1] and by using our molecular beam epitaxy system, respectively. The pink, gray and red squares represent the samples in this study, Ref. 2 and Ref. 3, respectively.

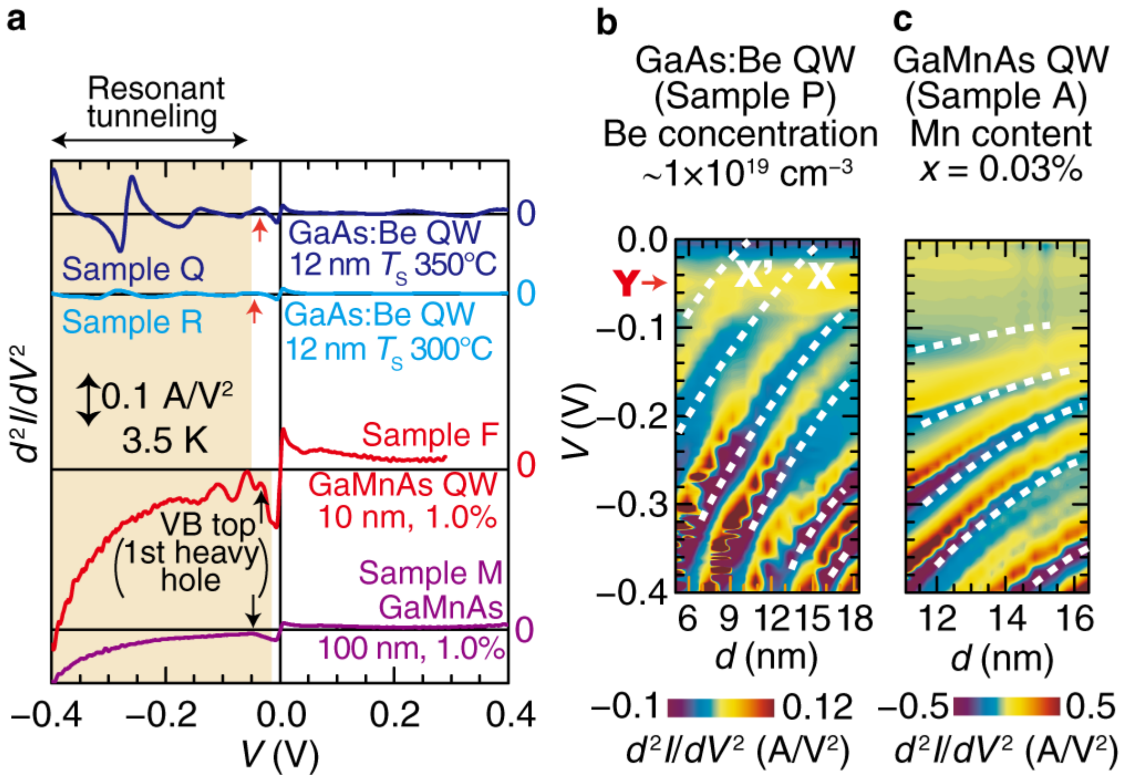

Supplementary Figure 4 Results of the reference experiments to confirm that the d^2I/dV^2-V oscillations observed in the negative-bias region in the samples with a GaMnAs quantum well are induced by the resonant tunneling levels in the quantum well. (**a**) d^2I/dV^2-V characteristics in Samples Q and R, each of which has a GaAs:Be quantum well (QW), and the ones in Samples F and M, each of which has a GaMnAs (QW) layer. The oscillations observed in the orange region are induced by resonant tunneling in the QW layer. (**b**,**c**) d^2I/dV^2-V characteristics represented by colour as a function of the QW thickness d in Sample P and Sample A, which have a GaAs:Be QW (the Be concentration is 1×10^{19} cm^{-3}) and a $Ga_{1-x}Mn_xAs$ QW (the Mn concentration x = 0.03%), respectively. In **b**, the oscillation dips labeled X and X', which are induced by resonant tunneling in the GaAs:Be QW, intersect with peak Y at d = ~8 nm and d = ~13 nm, respectively, and move toward the zero-bias position with increasing d. This result indicates that the Fermi level is located in the valence band (VB) in the heavily Be-doped GaAs. This feature is completely different from that of Sample A (**c**), in which the Fermi level is located in the band gap. (The data for Sample F are the same as those presented in ref. 4.)

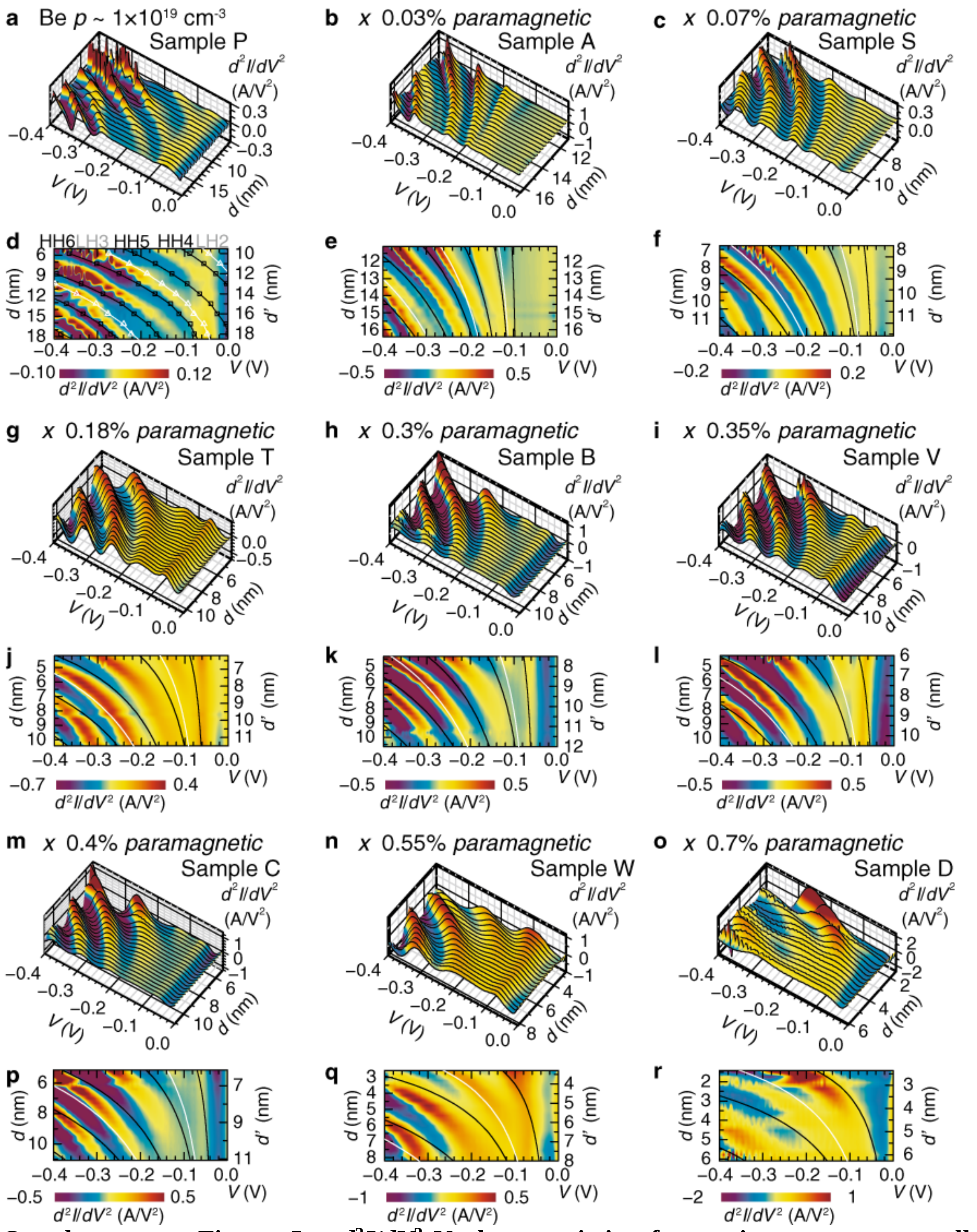

Supplementary Figure 5 **d^2I/dV^2-V characteristics for various quantum well thickness of GaAs:Be and of GaMnAs with the Mn concentration from 0.03% to 0.7%.** (**a-c**,**g-i**,**m-o**) Black solid curves are the d^2I/dV^2-V characteristics of the individual devices in Samples P, A, S, T, B, V, C, W and D at 3.5 K, respectively. (**d-f**,**j-l**,**p-r**) Comparison of the resonant dip voltages and calculated resonant levels. The black and white dots and curves indicate the calculated heavy hole (HH) and light hole (LH) resonant dips, respectively. We slightly modified the quantum well thickness *d* to *d'* so that the calculated resonant levels can be fit to the d^2I/dV^2 dip voltages as well as possible. The colour represents the d^2I/dV^2 intensity obtained by interpolating the measured values. Note that the range of *d*, *d'* and the scale of d^2I/dV^2 are different between the graphs.

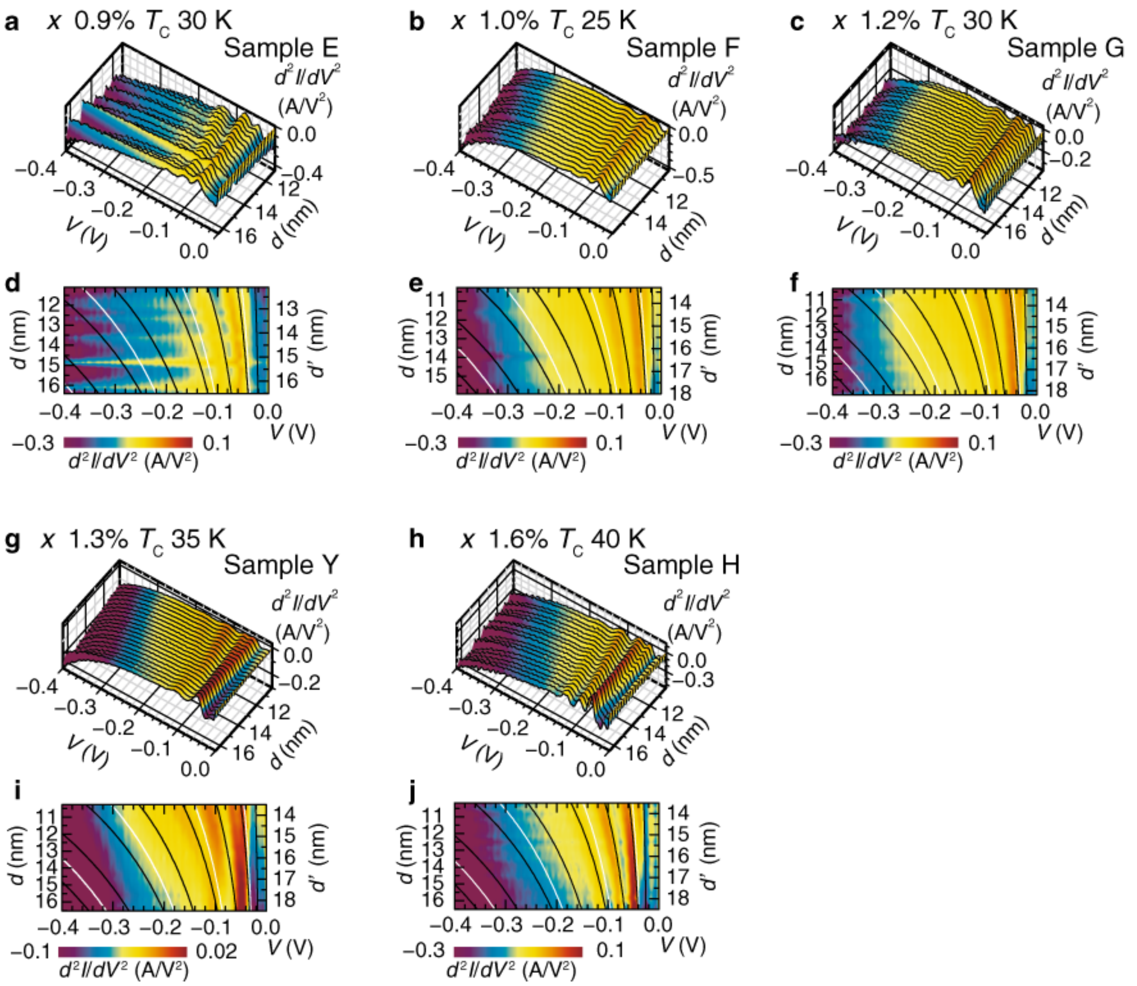

Supplementary Figure 6 d^2I/dV^2-V characteristics for various quantum well thicknesses of GaMnAs with the Mn concentration from 0.9% to 1.6%. (**a-c**,**g**,**h**) Black solid curves are the d^2I/dV^2-V characteristics of the individual devices in Samples E-G, Y and H at 3.5 K, respectively. (**d-f**,**i**,**j**) Comparison of the resonant dip voltages and calculated resonant levels. The black and white curves indicate the calculated heavy hole and light hole resonant dips, respectively. We slightly modified the quantum well thickness *d* to *d'* so that the calculated resonant levels can be fit to the d^2I/dV^2 dip voltages as well as possible. The colour represents the d^2I/dV^2 intensity obtained by interpolating the measured values. Note that the range of *d*, *d'* and the scale of d^2I/dV^2 are different between the graphs.

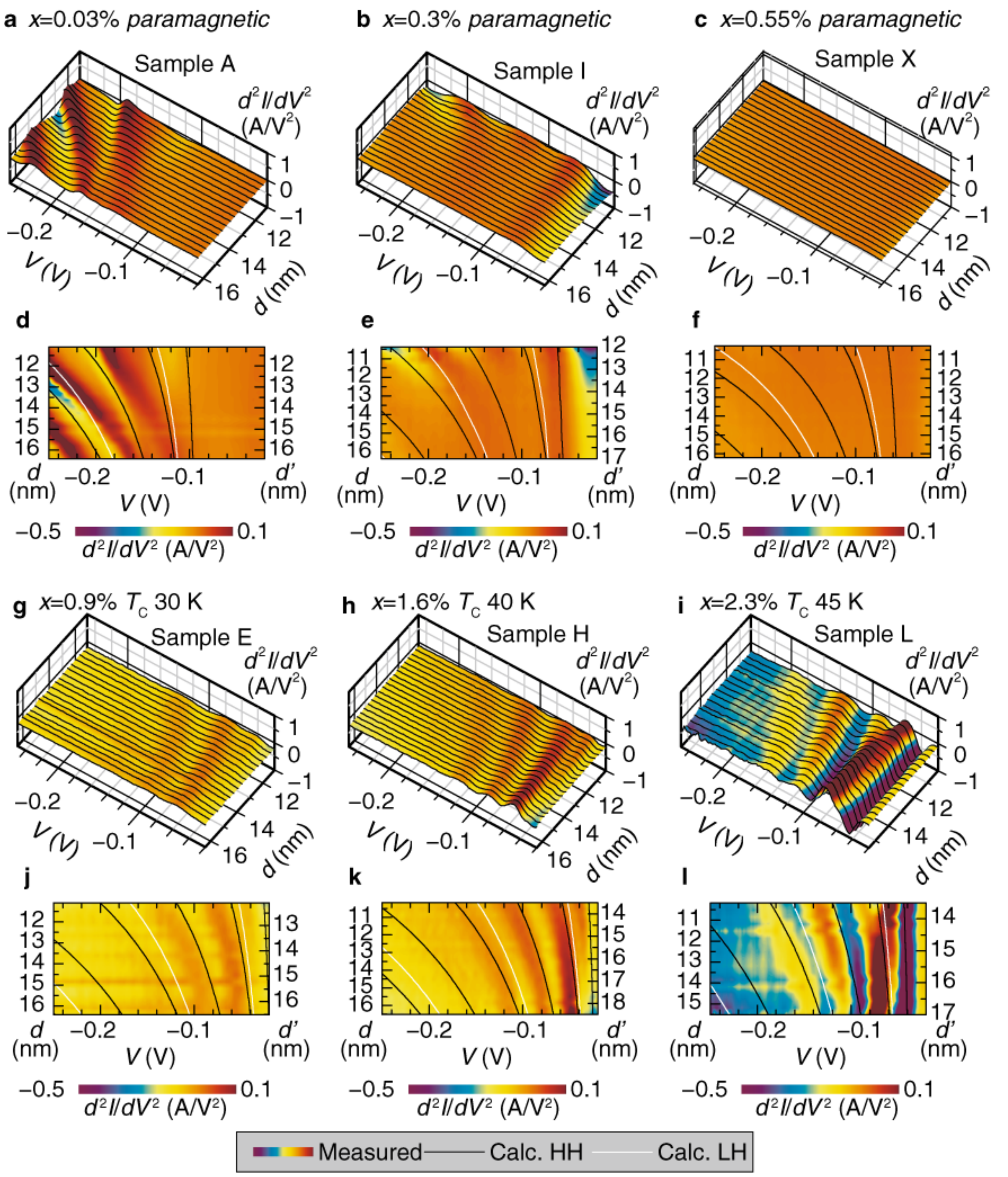

Supplementary Figure 7 d^2I/dV^2-V **characteristics for various quantum well thicknesses in Samples A, I, X, E, H and L.** (**a-c**,**g-i**) Black solid curves are the d^2I/dV^2-V characteristics of Samples A, I, X, E, H and L at 3.5 K, respectively. (**d-f**,**j-l**) Comparison of resonant dip voltages and calculated resonant levels. The black and white curves indicate the calculated heavy hole (HH) and light hole (LH) resonant dips, respectively. We slightly modified the quantum well thickness d to d' so that the calculated resonant levels can be fit to the d^2I/dV^2 dip voltages as well as possible. The colour represents the d^2I/dV^2 intensity obtained by interpolating the measured values.

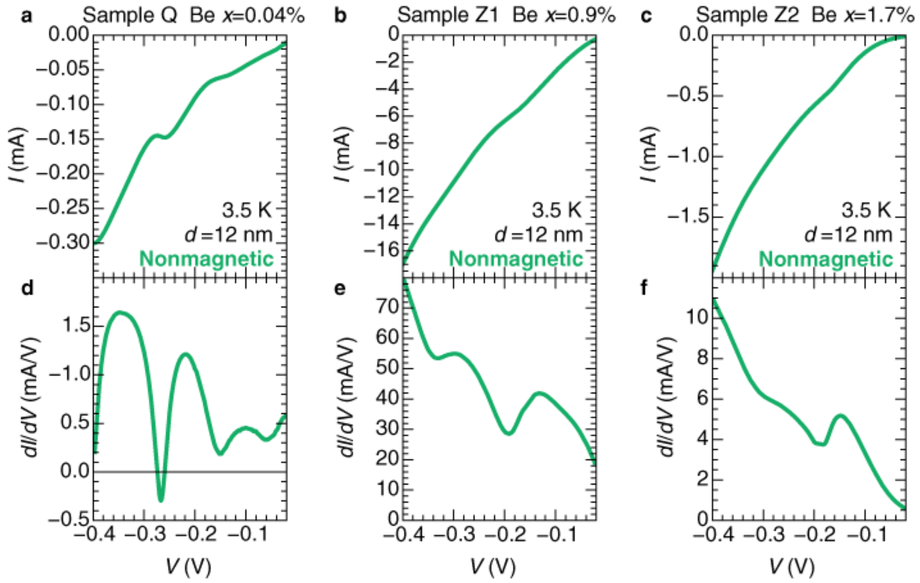

Supplementary Figure 8 Comparison of the *I*-*V* and *dI*/*dV*-*V* characteristics among the samples with a nonmagnetic GaAs:Be quantum well with a different doping concentration of Be. (**a**-**c**) *I*-*V* characteristics measured on Samples Q (the Be concentration x = 0.04%, the quantum well thickness d = 12 nm) (**a**), Z1 (x = 0.9%, d = 12 nm) (**b**) and Z2 (x = 1.7%, d = 12 nm) (**c**). (**d**-**f**) *dI*/*dV*-*V* characteristics derived from the *I*-*V* characteristics of Samples Q (**d**), Z1 (**e**) and Z2 (**f**).

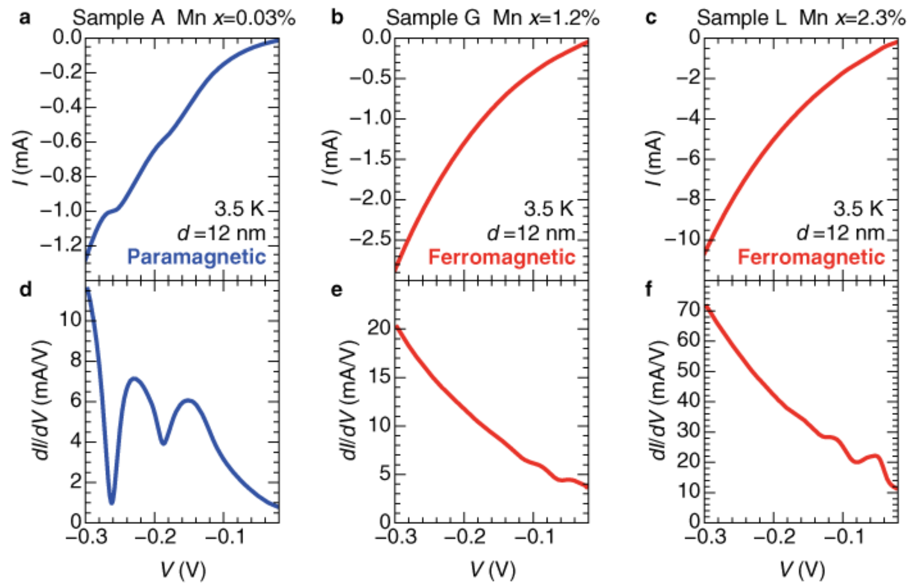

Supplementary Figure 9 Comparison of the *I*-*V* and *dI*/*dV*-*V* characteristics among the samples with a GaMnAs quantum well with a different doping concentration of Mn. (**a**-**c**) *I*-*V* characteristics measured on Samples A (the Mn concentration x = 0.03%, paramagnetic, the quantum well thickness d = 12 nm) (**a**), G (x = 1.2%, ferromagnetic, d = 12 nm) (**b**) and L (x = 2.3%, ferromagnetic, d = 12 nm) (**c**). (**d**-**f**) *dI*/*dV*-*V* characteristics derived from the *I*-*V* characteristics of Samples A (**d**), G (**e**) and L (**f**).

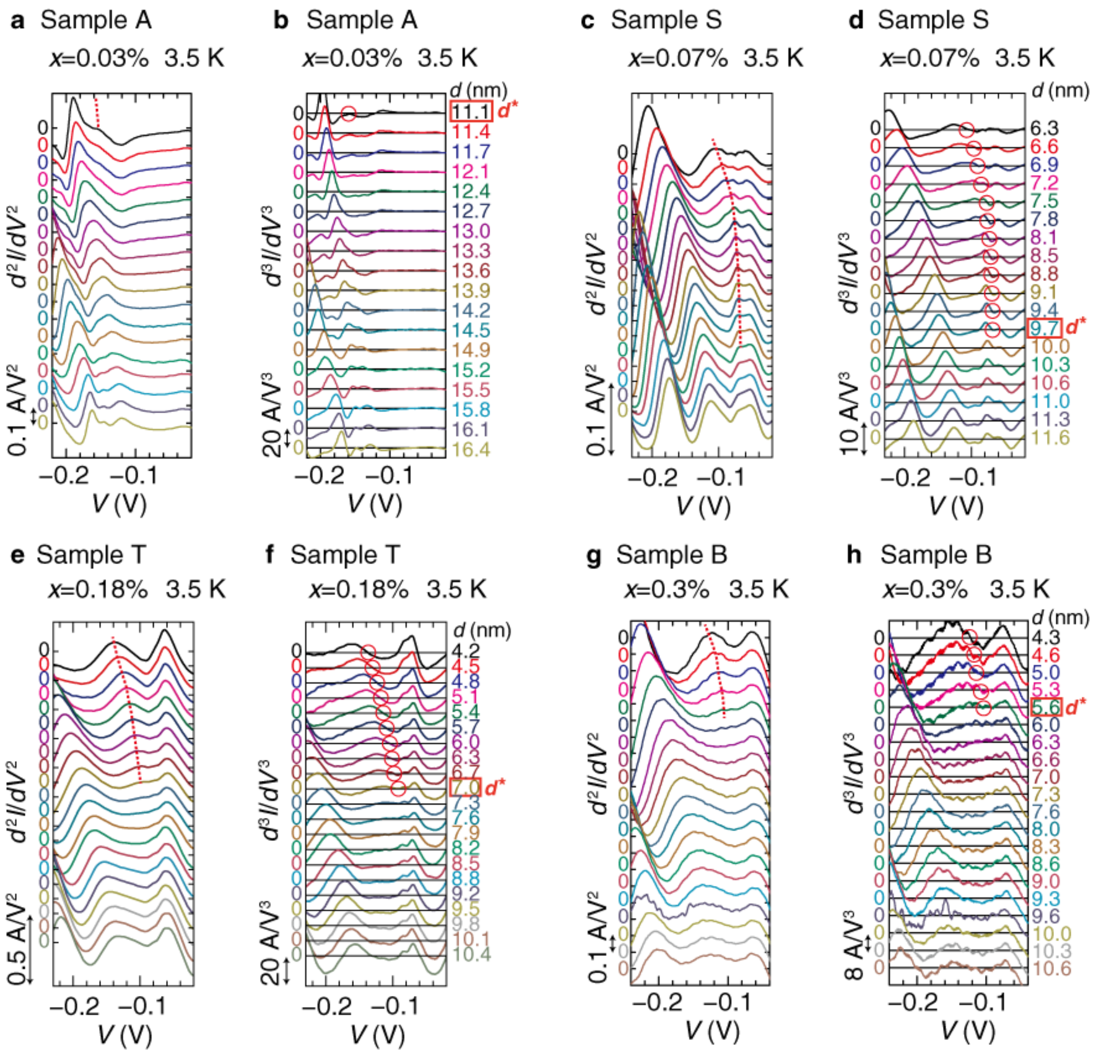

Supplementary Figure 10 Estimation of d^* values for the samples with the Mn concentration from 0.03% to 0.3%. (**a**,**c**,**e**,**g**) Resonant tunneling spectroscopy data of d^2I/dV^2-V characteristics in the paramagnetic Samples A (the Mn concentration x = 0.03%), S (x = 0.07%), T (x = 0.18%) and B (x = 0.3%). (**b**,**d**,**f**,**h**) Derived d^3I/dV^3-V characteristics in the paramagnetic Samples A, S, T and B. We define d^* as the quantum well thickness d value at which the peak between the dips of the first heavy and light hole levels disappears.

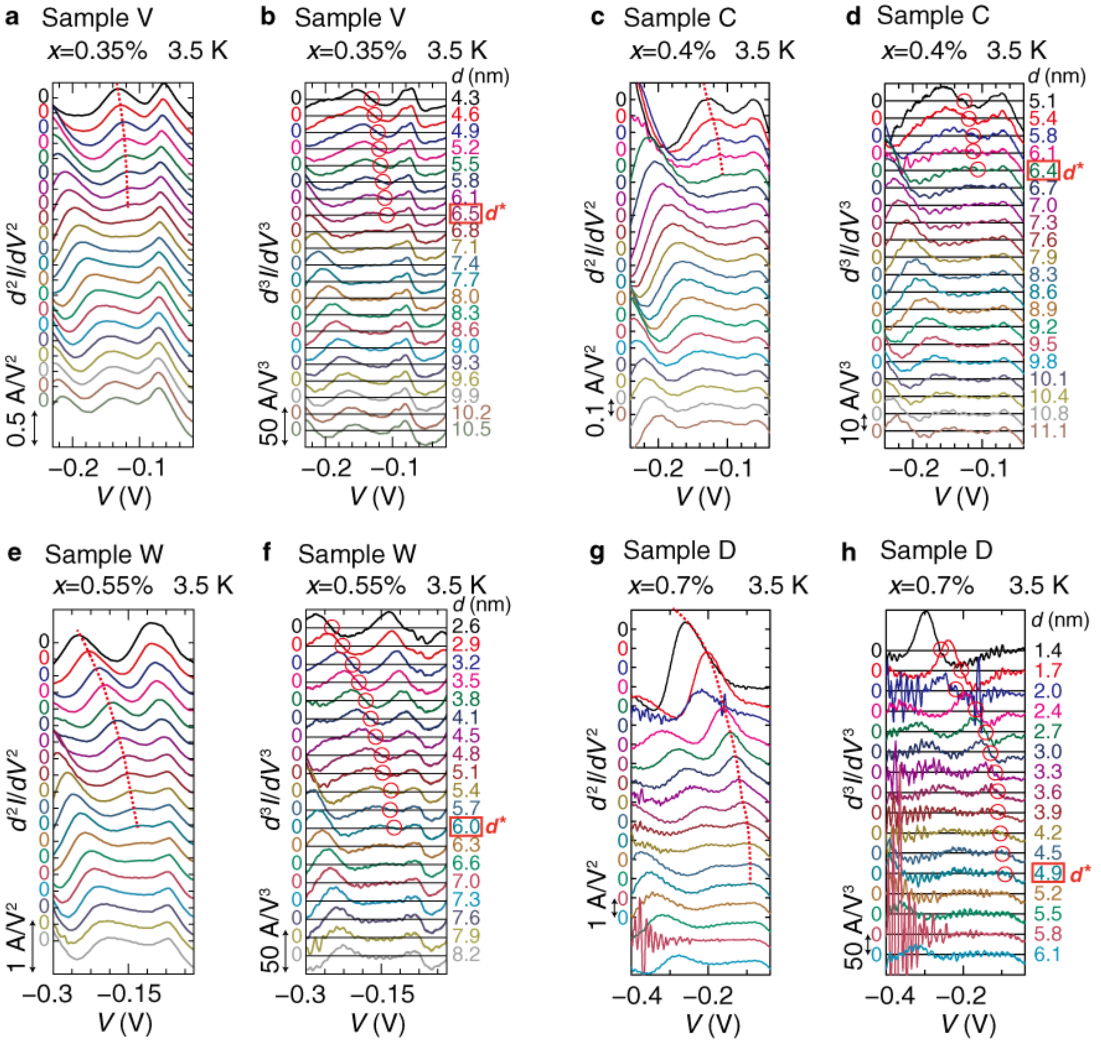

Supplementary Figure 11 Estimation of d^* values for the samples with the Mn concentration from 0.35% to 0.7%. (**a**,**c**,**e**,**g**) Resonant tunneling spectroscopy data of d^2I/dV^2-V characteristics in the paramagnetic Samples V (the Mn concentration x = 0.35%), C (x = 0.4%), W (x = 0.55%) and D (x = 0.7%). (**b**,**d**,**f**,**h**) Derived d^3I/dV^3-V characteristics in the paramagnetic Samples V, C, W and D. We define d^* as the quantum well thickness d value at which the peak between the dips of the first heavy and light hole levels disappears.

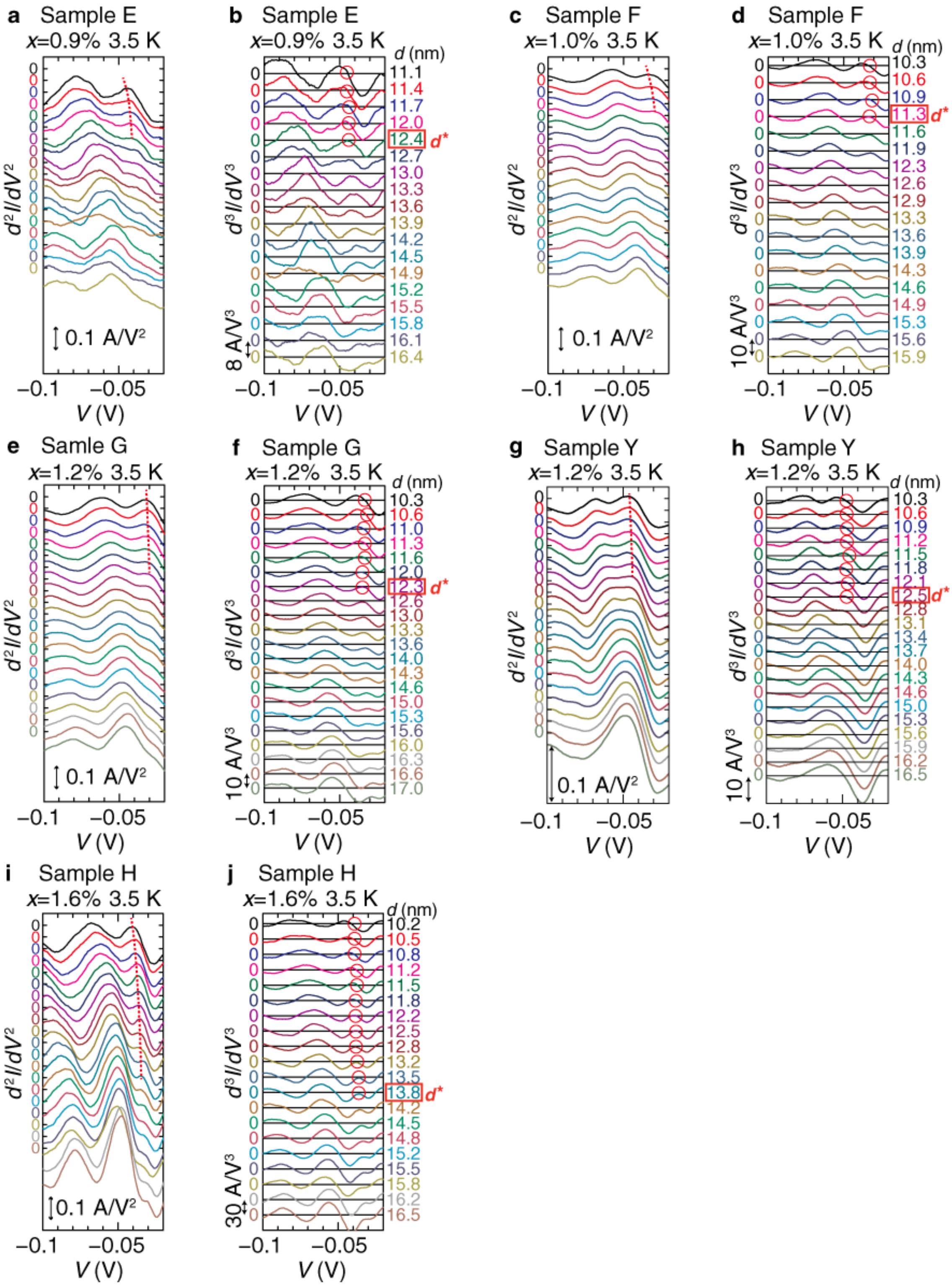

Supplementary Figure 12 Estimation of d^* values for the samples with the Mn concentration 0.9% to 1.6%. (**a**,**c**,**e**,**g**,**i**) Resonant tunneling spectroscopy data of the d^2I/dV^2-V characteristics in the ferromagnetic Samples E-G, Y and H. (**b**,**d**,**f**,**h**,**j**) Derived d^3I/dV^3-V characteristics in the ferromagnetic Samples E-G, Y and H. We define d^* as the quantum well thickness d value at which the peak between the dips of the first heavy and light hole levels disappears.

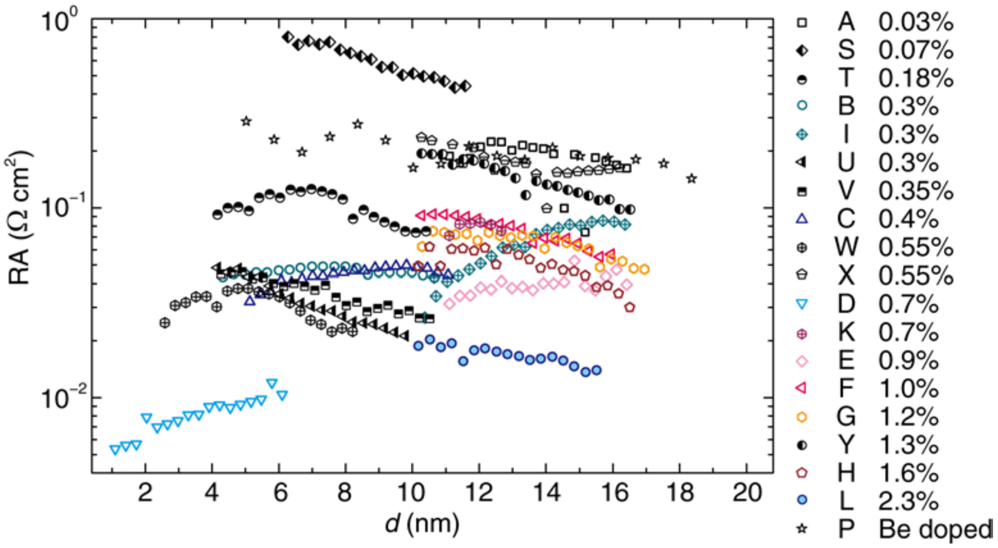

Supplementary Figure 13 Resistance-area product as a function of *d* for the devices examined in this study. Resistance-area product (*RA*) is measured for the devices with the various quantum well thickness *d* at the applied bias voltage V = -0.1 V at 3.5 K.

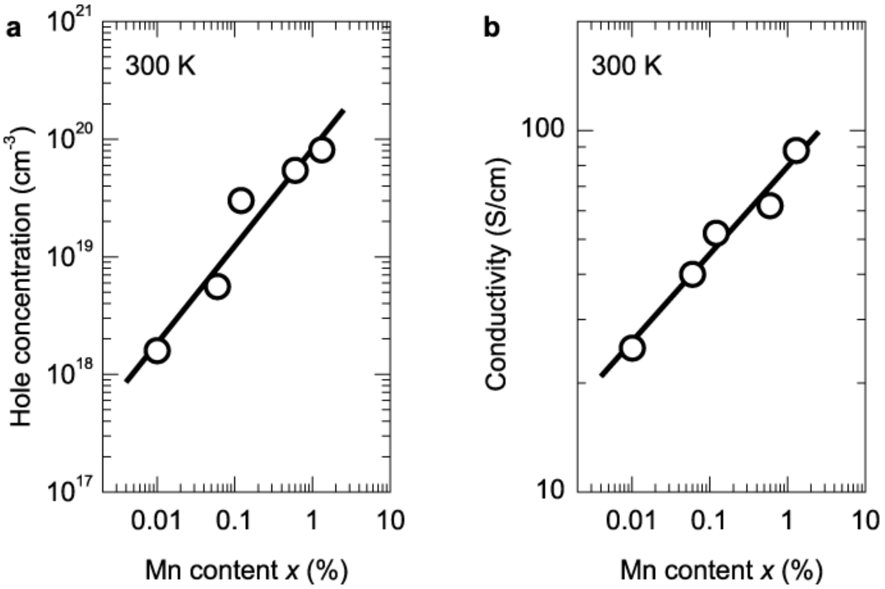

Supplementary Figure 14 Hole concentration and conductivity measured on 100-nm-thick $Ga_{1-x}Mn_xAs$ layers. (**a**) Hole concentration as a function of the Mn concentration *x*. (**b**) Conductivity as a function of *x*.

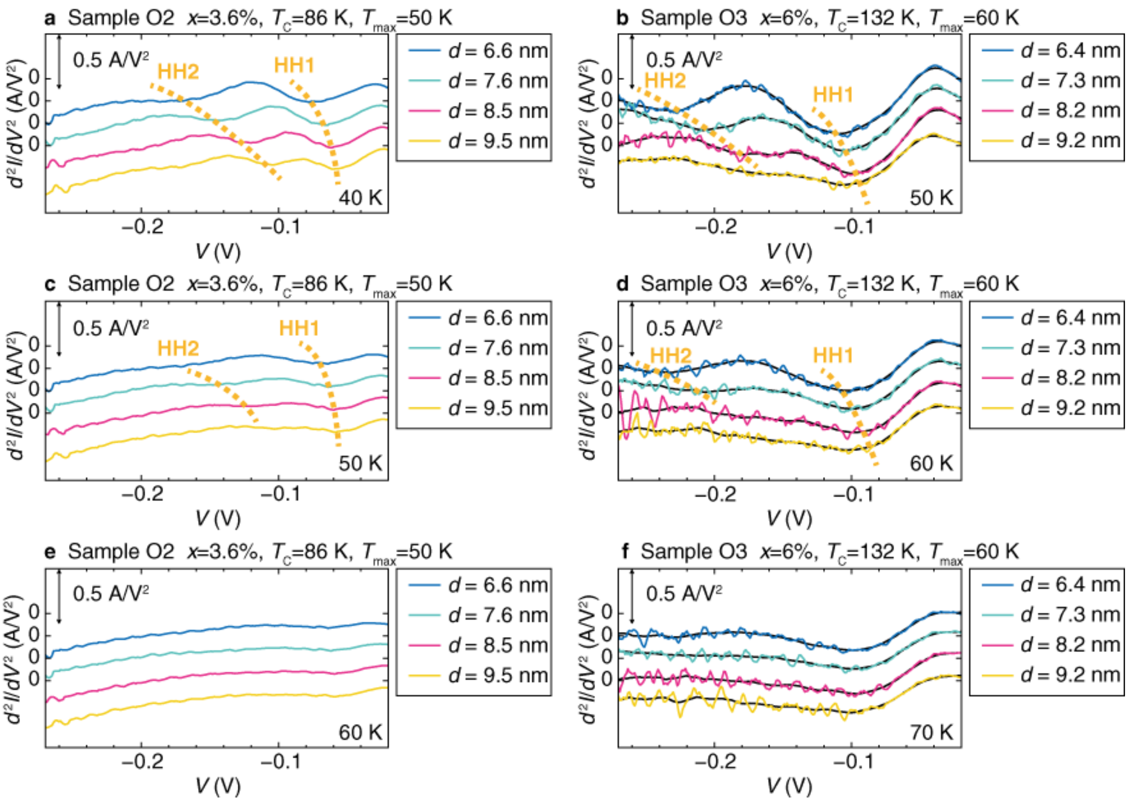

Supplementary Figure 15 Comparison of the d^2I/dV^2-V characteristics measured at different temperatures. (**a**,**c**,**e**) The d^2I/dV^2-V characteristics of Sample O2 (the Mn concentration x: 3.6%, the Curie temperature T_C: 86 K, T_{max}: 50 K) at 40 K (**a**), 50 K (**c**) and 60 K (**e**). We define T_{max} as the maximum temperature at which we can observe resonant tunneling with multiple dips in the d^2I/dV^2-V curves. The blue, green, pink and yellow curves represent the d^2I/dV^2-V characteristics of the devices with the quantum well thickness d = 6.6 nm, 7.6 nm, 8.5 nm and 9.5 nm, respectively. (**b**,**d**,**f**) The d^2I/dV^2-V characteristics of Sample O3 (x: 6%, T_C: 132 K, T_{max}: 60 K) at 50 K (**b**), 60 K (**d**) and 70 K (**f**). The blue, green, pink and yellow curves represent the d^2I/dV^2-V characteristics of the devices with d = 6.4 nm, 7.3 nm, 8.2 nm and 9.2 nm, respectively. The black solid curves represent the d^2I/dV^2-V characteristics derived from the Savitzky-Golay method with smoothing strongly (31 window size). The orange dotted lines represent the trace of the resonant dips of the first and second heavy hole (HH) levels.

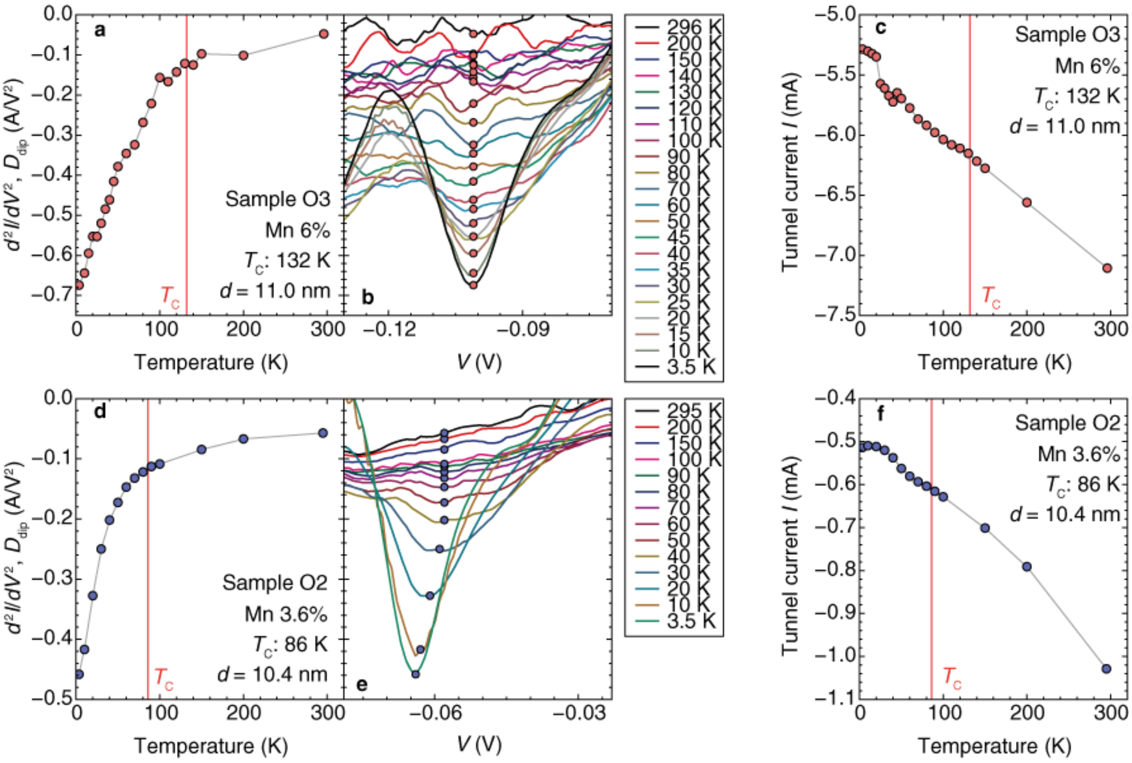

Supplementary Figure 16 Temperature dependence of the d^2I/dV^2-V and I. (**a**,**d**) Temperature T dependence of the d^2I/dV^2 values indicated by the dots in **b**,**e** in Sample O3 (the Mn concentration x: 6%, the Curie temperature T_C: 132 K, the quantum well thickness d: 11.0 nm) (**a**) and Sample O2 (x: 3.6%, T_C: 86 K, d: 10.4 nm) (**d**). T is ranging from 3.5 K to room temperature. (**b**,**e**) d^2I/dV^2-V characteristics in the applied bias voltage V region around the first heavy hole (HH) dip at various temperatures. The dots correspond to the HH1 dips at T ranging from 3.5 K to room temperature. (**c**,**f**) T dependence of the tunnel current I at the HH1 dip in **b**,**e**.

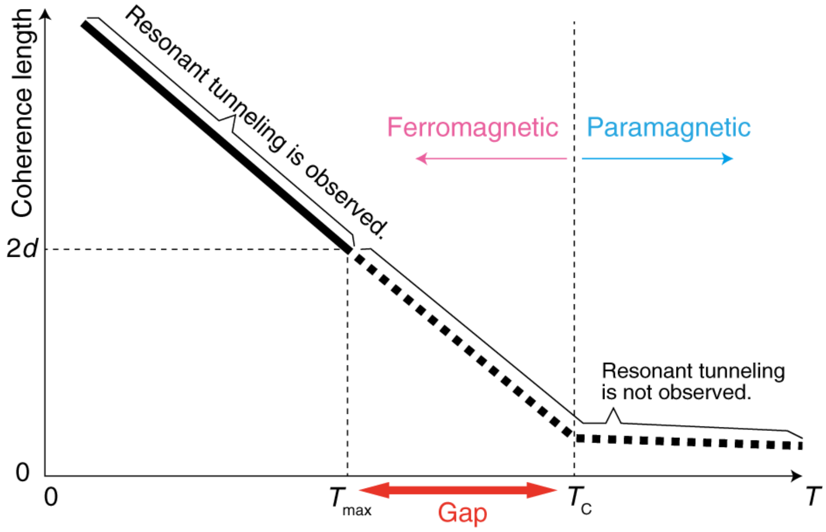

Supplementary Figure 17 Schematic diagram of the temperature (T) dependence of the coherence length of the valence band holes in GaMnAs. The solid and dotted lines represent the states that the resonant tunneling is observed below T_{max} and not observed above T_{max}, respectively. We define T_{max} as the maximum temperature at which we can observe resonant tunneling with multiple dips in the d^2I/dV^2-V curves. As the temperature T decreases, the coherence length starts to increase at the Curie temperature T_C. Resonant tunneling appears when the coherence length reaches twice the length of the quantum well thickness d.

Supplementary Table 1 Details of the samples with the GaAs:Be quantum well with the different Be concentration. The Be contents x of the $Ga_{1-x}Be_xAs$ quantum well (QW), the Mn contents y of the top $Ga_{1-y}Mn_yAs$ electrode, the QW thickness d and the growth temperature T_S of the QW layer.

Sample	Structure	x (%)	y (%)	d (nm)	T_S (°C)
Q	double barrier	Be 0.04	4	12	350
Z1	double barrier	Be 0.9	4	12	280
Z2	double barrier	Be 1.7	4	12	250

Supplementary Note 1

d^2I/dV^2-V characteristics in the positive bias region and the sharp change in d^2I/dV^2 at the zero bias.

Although the d^2I/dV^2 oscillations are clearly seen in the negative bias region, they are not clear in the positive bias region except in Sample Q. As explained in our previous publications[5,6], when holes are injected from the GaAs:Be electrode (negative bias) with a hole concentration of ~1×10^{18} cm^{-3} which has a small Fermi surface, tunneling occurs in the small in-plane wave-number ($k_{||}$) region. Because the subbands formed by the quantum-size effect in the quantum well (QW) have large energy separation near the Γ point, the tunneling holes with small $k_{||}$ can detect the resonant levels separately in the GaMnAs QW. On the other hand, when holes are injected from the GaMnAs layer, which has a large Fermi surface, tunneling occurs in the large $k_{||}$ region and holes with large $k_{||}$ detect many resonant levels at the same time owing to the dispersion of the subbands. In this case, it is difficult to detect each level clearly.

Also, the growth temperature T_S of QW is related to the strength of the resonant tunneling effect in the QW. As shown in Supplementary Fig. 4, in Sample Q with a nonmagnetic GaAs:Be QW grown at 350°C, we can see small oscillations induced by the resonant tunneling levels in QW even in the positive bias region. However, in Sample R with a GaAs:Be QW grown at 300°C, the oscillation is not seen clearly in the positive bias region. This is because the crystal quality of the QW in Sample Q is better than that in Sample R.

The large jump in the d^2I/dV^2-V curves at the zero bias in our samples (Supplementary Fig. 4a) has been frequently observed in GaMnAs heterostructures in studies by other groups. This jump, which is called zero-bias anomaly, is considered to be induced by the Altshuler-Aronov correlation gap in GaMnAs[7]. However, this is not relevant in the present study.

Supplementary Note 2
Quality of our samples and stability of our device fabrication process

It is known that the concentration of the crystal defects, such as As anti-sites and Mn interstitials, and the surface morphology are changed by T_S of the GaMnAs layer. Higher T_S is better for the crystal quality as long as the MnAs clustering does not occur. (The MnAs clustering can be easily distinguished by the observation of reflection high-energy electron diffraction.) Thus, we used nearly the highest limit of T_S at each Mn concentration x (see Supplementary Figs 1b and 3). Actually, the Curie temperature T_C - x line obtained in our group is almost the same as that obtained in the systematic and detailed study on high-quality GaMnAs films reported by Jungwirth *et al.*[1] as shown in Supplementary Fig. 3. Therefore, we think that the quality of our samples is among the best reported so far.

As can be seen in each colour coded map of d^2I/dV^2 as a function of V and d (Supplementary Figs 5-7), in which each d^2I/dV^2-V curve corresponds to a different device, the data look very continuous as a function of d. This indicates that our device fabrication process is stable and does not influence the experimental results.

Supplementary Note 3
Influence of growth temperature dependence on the resonant tunneling effect

To examine the influence of the difference in T_S of the GaMnAs QW layer on the resonant tunneling effect, we compare the d^2I/dV^2-V characteristics between Samples B and U, in which the GaMnAs QW layer was grown at different T_S = 330 °C and 265 °C, respectively, and has the same Mn content x=0.3%. It is obvious that lowering T_S results in weaker oscillations of the d^2I/dV^2-V characteristics, as shown in Supplementary Fig. 2. In Sample U, the first heavy hole (HH) dip is not observed even at the smallest d (4.3 nm). Thus, d^* of Sample U is estimated to be less than 4.3 nm, which is shorter than the d^* value of Sample B (5.6 nm). We used nearly the highest limit of T_S for the growth of the GaMnAs QW to obtain a high quality QW in all samples except for Sample U (see Supplementary Note 2).

Supplementary Note 4
Experimental evidence that the d^2I/dV^2 oscillations originate from the resonant tunneling levels in the GaMnAs quantum wells

We confirm that the d^2I/dV^2 oscillations observed in the GaMnAs-QW heterostructures are not induced by the resonant levels formed in the triangular potential region at the AlAs/GaAs:Be interface[8] or by the diffused Mn or Be atoms in the AlAs barriers, but are definitely induced by the resonant levels formed in the GaMnAs QW. In Supplementary Fig. 4a, we compare the d^2I/dV^2-V curves of Samples Q and R, each of which has a GaAs:Be QW, and those of Samples F and M, each of which has a GaMnAs QW. We focus only on the negative-bias region, where clear oscillations are observed. In all the d^2I/dV^2-V curves presented in Supplementary Fig. 4a, we observe a small peak at approximately -0.05 - -0.02 V, indicated by a red or black arrow. For the GaAs:Be QW (Samples Q and R), the origin of this peak (red arrow) is unknown, but it is most likely related to an extrinsic effect such as the state induced by the Be dopant atoms in the QW or the state related to the GaMnAs top electrode because this peak does not move when d changes, as shown later (see peak Y in Supplementary Fig. 4b). In Samples F and M, however, this peak (black arrow) must have a different origin because the peak moves with changes in d (ref. 4). This peak is attributed to HH1 in Sample F and to the valence band (VB) top energy of the GaMnAs QW in Sample M. Note that these origins are essentially the same because HH1 is formed by the quantization of the VB. Meanwhile, in the $V < -0.05$ V region of all d^2I/dV^2-V curves in Supplementary Fig. 4a, the intensity of the oscillations clearly depends on the properties of the QW, such as d or the crystal quality. For instance, the oscillations are much clearer in Sample Q than in Sample R; this result is consistent with the fact that the QW layer in Sample Q is of better crystal quality than that in Sample R because of the higher T_S of 350°C used for Sample Q. Next, we compare the data between Samples F and M, which have the same GaMnAs heterostructure but different d of 10 nm and 100 nm, respectively. In Sample F, we observe clear oscillations, whereas we do not observe any clear oscillations when $V < -0.05$ V in Sample M because of the much weaker confinement of holes in the thicker GaMnAs QW of Sample M. These results indicate that the oscillation observed in the negative-bias region, except for the sharp increase at zero bias, originates from the GaMnAs QW in Sample F. The intensity of the resonant states formed at the AlAs/GaAs:Be interface, if any, would not be changed by varying d or T_S. The diffusion of impurity atoms may depend on T_S; however, there is a clear difference between the results of Samples F and M even though nearly the same T_S was used for the QW growth for both samples. Thus, the diffusion of the impurity atoms is not related to the d^2I/dV^2 oscillations observed in this study.

Furthermore, we confirm that a change in the Fermi-level position in the QW leads

to a clear difference in our experimental results, as expected from the principle of our resonant tunneling spectroscopy measurements presented in Fig. 2d and 2f. We measured the d^2I/dV^2-V characteristics of Sample P, which has a degenerate GaAs:Be QW with various d (Supplementary Fig. 4b). Because the Fermi-level position and the band structure of GaAs:Be are well known, these measurements are a good test to verify that our method performs well in investigating the band structures of the QW. In nearly all the d^2I/dV^2-V curves, we observe clear oscillations whose bias-voltage position strongly depends on d, which are induced by resonant tunneling in the QW and are indicated by the white dotted curves based on the calculation of the resonant levels (Supplementary Fig. 5d). We also observe a peak (labeled Y, see Supplementary Fig. 4b) at approximately -0.05 to -0.02 V, which does not depend on d and has the same origin as the peaks indicated by the red arrows in Supplementary Fig. 4a. As shown in Supplementary Fig. 4b, the oscillation dips labeled X and X', which are induced by resonant tunneling in the QW, intersect with peak Y at d = ~8 nm and d = ~13 nm, respectively, and move toward the zero-bias position with increasing d. This result indicates that the resonant levels do not converge in the negative-bias region and is consistent with the fact that the Fermi level is located in the VB in the heavily Be-doped GaAs QW of Sample P, where the Fermi-level position is estimated to be ~40 meV below the top of the VB. This feature is completely different from that of Sample A (Supplementary Fig. 4c), in which the resonant levels converge in the negative-bias region and thus the Fermi level is located in the band gap. Based on these results, we conclude that all d^2I/dV^2 oscillations observed in the negative-bias region in our devices with GaMnAs QWs are induced by resonant tunneling in the QW, with the exception of the sharp feature at zero bias (the so-called zero-bias anomaly)[2,4-6,9].

Supplementary Note 5
Fitting by theoretical calculations of the resonant levels

In Supplementary Figs 5-7, the black and white curves in the two-dimensional maps show the calculated resonant levels of the HH and light-hole (LH) sub-bands, respectively. Since the energy relative to the Fermi level has a linear relation with V in the tunnel experiments[10], the resonant levels are represented by $s(E_n + E_\Delta)$. Here, s is the total voltage divided by the voltage applied to the bottom AlAs tunnel barrier, E_n is the energy of the nth levels of the HH and LH sub-bands in the $Ga_{1-x}Mn_xAs$ QW with respect to the bulk VB top energy, E_Δ is the energy between the Fermi level and the bulk VB top. Note that the range of d is different among the samples in Supplementary Figs 5 and 6. Thus, direct comparison of the intensity of resonant tunneling may be difficult in these graphs.

In the calculations, we set s=2.0 because we used the same AlAs barriers for the bottom and upper tunneling barrier layers.[10] We calculated E_n as a function of d by using the 4×4 Luttinger-Kohn $\boldsymbol{k \cdot p}$ Hamiltonian[11] and the transfer matrix method.[10] We slightly modified d to d' so that the calculated resonant levels can be fit to the d^2I/dV^2 dip voltages as well as possible. Here, d' is the thickness of the $Ga_{1-x}Mn_xAs$ QW used in the calculation of the resonant levels.

Supplementary Note 6
Resonant tunneling effect in the nonmagnetic GaAs:Be quantum wells as a function of the Be doping concentration

As shown below, in the case of the Be doped GaAs QW, the behavior of the resonant tunneling is quite standard; the resonant tunneling monotonously becomes weaker as the Be concentration is increased. This is quite different from the case of GaMnAs.

We fabricated three Samples Q, Z1 and Z2 with a Be–doped GaAs QW consisting of $Ga_{0.96}Mn_{0.04}As$ (20 nm) / AlAs (6 nm) / $Ga_{1-x}Be_xAs$ QW (12 nm) / AlAs (6 nm) / GaAs:Be (100 nm, p=1×10^{18} cm^{-3}) on a p^+-GaAs (001) substrate, in which the doping concentration x of Be in the QW is 0.04%, 0.9% and 1.7%, respectively (see Supplementary Table 1). The *I*-*V* and *dI*/*dV*-*V* characteristics have oscillations induced by resonant tunneling in the $Ga_{1-x}Be_xAs$ QW (Supplementary Fig. 8). The oscillation amplitude monotonously decreases as the Be doping level increases. This means that the doping of Be weakens the VB ordering of the $Ga_{1-x}Be_xAs$ QW. This is clearly different from the case of GaMnAs, in which the VB ordering is restored when the Mn concentration is larger than 1% (see Supplementary Fig. 9). These results clearly indicate that the restoration of the VB ordering requires the ferromagnetic ordering in the QW.

Supplementary Note 7
Estimation of d^*

We defined d^* as the d value at which the peak between the dips of the HH1 and the LH1 in the d^2I/dV^2-V curves disappears with increasing d due to the weakening of the quantum confinement. We derived d^3I/dV^3 as the auxiliary to judge the disappearance of the d^2I/dV^2 peak. At a d^2I/dV^2 peak, the sign of d^3I/dV^3 changes from positive to negative. We show the d^2I/dV^2-V and d^3I/dV^3-V characteristics of the paramagnetic samples (x = 0.03% - 0.7%) in Supplementary Figs 10,11 and the ones of the ferromagnetic samples (x = 0.9% - 1.6%) in Supplementary Fig. 12. The red dotted curves trace the d^2I/dV^2 peaks between the HH1 and LH1 dips. The red circles indicate the points of the sign change of d^3I/dV^3 corresponding to the d^2I/dV^2 peaks between the HH1 and LH1 dips.

Supplementary Note 8
Resistance-area product values of our diodes devices

In Supplementary Fig. 13, we show the resistance-area product (*RA*) values of our diode devices measured at V = -0.1 V at 3.5 K. One may think that the *RA* difference between the devices may influence the intensity of the oscillations in the d^2I/dV^2-V curves, but we cannot see any clear correlations between the *RA* values and the d^2I/dV^2-V characteristics (either the bias voltages corresponding to the resonant levels or the intensities of the d^2I/dV^2 oscillations).

Supplementary Note 9
Data of the hole concentration and the conductivity as a function of *x*

In Supplementary Fig. 14, we show the hole concentration and the conductivity data as a function of x measured by the Hall measurements in the reference samples with a single 100-nm-thick GaMnAs layer grown in a similar condition as that for the GaMnAs QW. We do not see any sharp changes near x=0.9%, which means that the observed sharp change of the band ordering is not due to the fluctuation of the sample growth conditions. Therefore, the sudden restoration of the VB ordering presented in this study is an intrinsic feature of GaMnAs. In addition, we have shown that the Fermi level position of the samples having the GaMnAs QW moves continuously without any sharp changes with increasing x (ref. 4), but this x dependence of the Fermi level position is not directly related to the sudden restoration of the VB ordering.

Supplementary Note 10
Temperature dependence of the resonant tunneling

We measured the d and V dependences of d^2I/dV^2 at various temperature (T) ranging from 3.5 K to 200 K as shown in Fig. 4 in the main manuscript. We define T_{max} as the maximum temperature at which we can observe resonant tunneling with multiple dips in the d^2I/dV^2-V curves. As shown in Supplementary Fig. 15, T_{max} of Sample O2 is estimated to be 50 K because both the HH1 and HH2 dips are observed until 50 K, while T_{max} of Sample O3 is estimated to be 60 K because both the HH1 and HH2 dips are observed until 60 K.

We found that the T dependence of the intensity of the oscillation of the d^2I/dV^2-V curves induced by resonant tunneling has behavior related to T_C. Here, we define D_{dip} as the d^2I/dV^2 value at the HH1 dip. We plot the T dependence of D_{dip} in Samples O3

and O2 from 3.5 K to room temperature in Supplementary Fig. 16a,d. The dip positions are indicated by the dots on the d^2I/dV^2-V curves measured at various T shown in Supplementary Fig. 16b,e. The D_{dip}-T curves show a threshold at around $T = T_C$ in both samples with different T_C. This result suggests that T_C is a starting point of the increase in the coherence length of the VB holes in the QW as T decreases.

The T dependence of the tunnel current I at the HH1 dip does not have a notable change at around $T = T_C$ (Supplementary Fig. 16c,f). Thus, the threshold of the D_{dip}-T curve is not attributed to the T dependence of I.

As shown in Fig. 4d in the main manuscript, T_{max} and T_C show a positive correlation, but T_{max} is lower than T_C for all the samples. The possible reason is as follows. At the resonant tunneling condition, holes are required to make a round trip without scattering in the QW; holes must travel twice the length of the thickness (d) of the QW. As shown in Supplementary Fig. 17, the coherence length of the VB holes starts to increase at T_C as T decreases. Resonant tunneling appears when the coherence length reaches $2d$. This temperature corresponds to T_{max}. Thus, T_{max} is lower than T_C.

Supplementary References

1 Jungwirth, T. *et al*., supplementary material in Systematic study of Mn-doping trends in optical properties of (Ga,Mn)As. *Phys. Rev. Lett.* **105**, 227201 (2010).

2 Ohya, S., Takata, K. & Tanaka, M. Nearly non-magnetic valence band of the ferromagnetic semiconductor GaMnAs. *Nat. Phys.* **7**, 342-347 (2011).

3 Ohno, K., Ohya, S. & Tanaka, M. Properties of heavily Mn-doped GaMnAs with Curie temperature of 172.5 K. *J. Supercond. Nov. Magn.* **20**, 417-420 (2007).

4 Muneta, I., Terada, H., Ohya, S. & Tanaka, M. Anomalous Fermi level behavior in GaMnAs at the onset of ferromagnetism. *Appl. Phys. Lett.* **103**, 032411 (2013).

5 Ohya, S., Hai, P. N., Mizuno, Y. & Tanaka, M. Quantum size effect and tunneling magnetoresistance in ferromagnetic-semiconductor quantum heterostructures. *Phys. Rev. B* **75**, 155328 (2007).

6 Tanaka, M., Ohya, S. & Hai, P. N. Recent progress in III-V based ferromagnetic semiconductors: Band structure, Fermi level, and tunneling transport. *Appl. Phys. Rev.* **1**, 011102 (2014).

7 Altshuler, B. L. & Aronov, A. G. Zero bias anomaly in tunnel resistance and electron-electron interaction. *Solid State Commun.* **30**, 115-117 (1979).

8 Dietl, T. & Ohno, H. Dilute ferromagnetic semiconductors: Physics and spintronic structures. *Rev. Mod. Phys.* **86**, 187-251 (2014).

9 Ohya, S., Muneta, I., Xin, Y., Takata, K. & Tanaka, M. Valence-band structure of ferromagnetic semiconductor (In,Ga,Mn)As. *Phys. Rev. B* **86**, 094418 (2012).

10 Tsu, R. & Esaki, L. Tunneling in a finite superlattice. *Appl. Phys. Lett.* **22**, 562-564 (1973).

11 Wessel, R. & Altarelli, M. Resonant tunneling of holes in double-barrier heterostructures in the envelope-function approximation. *Phys. Rev. B* **39**, 12802-12807 (1989).
